# Handling missing data of using the XGBoost-based multiple imputation by chained equations regression method

**DOI:** 10.3389/frai.2025.1553220

**Published:** 2025-04-03

**Authors:** Zhao Jinbo, Li Yufu, Mo Haitao

**Affiliations:** ^1^Shangwan Coal Mine, Ejin Horo Banner, Ordos, China; ^2^CCTEG Xi’an Research Institute Co. Ltd., Xi’an, China

**Keywords:** mine ventilation parameters, XGBoost, multiple imputation, data completeness, intelligent management

## Abstract

This study introduces an XGBoost-MICE (Multiple Imputation by Chained Equations) method for addressing missing data in mine ventilation parameters. Using historical ventilation system data from Shangwan Coal Mine, scenarios with different missing rates (5, 10, and 15%) and iteration numbers (30 and 50) were simulated to validate the accuracy and effectiveness of the approach. The results demonstrate that as the missing rate increased from 5 to 15%, the Mean Squared Error (MSE) rose from 0.0445 to 0.3254, while the Explained Variance decreased from 0.988309 to 0.943267. Additionally, the Mean Absolute Error (MAE) increased by 0.29. Iteration experiments on the “frictional resistance per 100 meters” attribute showed convergence of MSE and MAE after six iterations. Overall, the XGBoost-MICE method exhibited high imputation accuracy and stable convergence across various missing data scenarios, providing robust technical support for optimizing intelligent mine ventilation systems.

## 1 Introduction

The mine ventilation system is a critical component in ensuring mine safety and operational efficiency (Sjöström et al., 2020; Liu et al., 2021; Zhou et al., 2023). Proper design of ventilation parameters effectively regulates airflow within the mine, reduces harmful gas concentrations, ensures miners’ safety, and improves productivity. With the development of intelligent mine management systems, data collection and monitoring technologies have been widely adopted, providing a wealth of real-time data. However, due to factors such as sensor failures and environmental interference, the issue of missing data in mine ventilation parameters has become increasingly prominent (Shriwas and Pritchard, 2020; Semin et al., 2020; Nardo and Yu, 2021). This not only affects the accuracy of data analysis and decision-making but also limits the optimization and adaptive adjustment of intelligent ventilation systems.

To address this problem, numerous data processing methods have been proposed to fill in missing data and restore data integrity. Traditional methods for missing data imputation, such as mean imputation and nearest-neighbor imputation, are simple and practical but often perform poorly when handling complex data, particularly when the data contain multiple missing values and strong correlations (Pujianto et al., 2019; Lalande and Doya, 2022). In the context of mine ventilation parameter imputation, some researchers have focused on aspects like ventilation friction resistance coefficients. For example, Xu et al. (2017) studied the calibration of mine ventilation network (MVN) models and proposed a nonlinear optimization-based calibration method. This method not only aligns simulated airflow distribution results with on-site measurements but also minimizes errors in other parameters. Applications in real-world cases demonstrated that the calibrated MVN model built from ventilation survey results and Ventsim software produced better airflow simulation results, validating the effectiveness of the calibration method. Similarly, Bosikov et al. (2023) investigated mine ventilation network parameters to ensure fire safety during coal mine development, proposing a novel modeling approach to enhance the control reliability of ventilation systems. Using probability theory and statistical methods, they conducted a comprehensive analysis of pneumatic processes in coal mines and applied spline interpolation to process spatial data. Their research developed a universal transfer function expression considering delay factors, describing the dynamic characteristics of mine ventilation parameters under different operating conditions. Hao et al. (2023) explored a multi-branch joint regulation method for mine ventilation based on sensitivity analysis, proposing a solution to address the issue of unmet airflow demand in specific branches under demand-based ventilation. By introducing the concept of sensitivity rate, they quantitatively analyzed the sensitivity variation patterns of branch airflow to multi-branch resistance and derived the calculation formula for sensitivity rate matrices. The Lagrange interpolation method was further employed to optimize the relationship between airflow sensitivity and resistance. Despite their contributions, these traditional methods often fail to meet the precision requirements for mine ventilation data processing, especially in real-time optimization scenarios, where more efficient and accurate algorithms are needed to handle missing data.

In recent years, machine learning-based approaches for missing data processing have gained increasing attention. Alkabbani et al. (2022) proposed a machine learning-based method for predicting air quality indices (AQI), combining multivariate data imputation techniques. Using artificial neural networks to predict hourly concentrations of PM2.5 and PM10, their approach extended to other air pollutants (e.g., O3, SO2, NO2, CO) for AQI estimation. They employed the missForest imputation method based on random forests, which significantly outperformed linear imputation methods, achieving an AQI prediction accuracy of 92.41%. Raja and Thangavel (2020) introduced an unsupervised machine learning method for handling missing values, based on coarse K-means centroids, and compared it with other imputation methods such as K-means centroids and fuzzy C-means centroids. Experimental analysis across benchmark datasets (e.g., Dermatology, Pima, Wisconsin, Yeast) validated the effectiveness of their proposed method in handling missing values. Lyngdoh et al. (2022) applied machine learning-based methods to predict concrete strength, demonstrating the use of data imputation techniques to enhance dataset completeness and improve predictions of concrete compressive and tensile strength. Their findings indicated that datasets imputed with k-Nearest Neighbor (kNN, 10 neighbors configuration) yielded the best results when paired with the Extreme Gradient Boosting (XGBoost) algorithm.

The XGBoost (Extreme Gradient Boosting) algorithm, known for its powerful predictive capabilities and exceptional performance in handling complex data, has shown great potential for missing data imputation. By constructing multiple decision trees, XGBoost effectively models high-dimensional data, enabling accurate predictions of missing values. Meanwhile, Multiple Imputation by Chained Equations (MICE), as a classic multiple imputation method, has been widely used for missing data processing. By generating multiple imputed datasets through iterative processes, MICE reduces the bias of traditional single-imputation methods and enhances the credibility of imputed results. Laqueur et al. (2022) proposed a SuperMICE method, integrating ensemble machine learning into MICE. This method employed the Super Learner algorithm to predict the conditional means of missing values and optimized model selection by combining local kernel estimates of variance. Getz et al. (2023) compared MICE, random forests, and denoising autoencoders for multiple imputation performance in electronic health records (EHR) data. Their study found that MICE and random forests exhibited lower bias under completely random missing mechanisms, whereas denoising autoencoders had higher bias. Under non-random missing mechanisms, all methods showed increased bias proportional to the extent of missing data. These studies highlight that the choice of imputation models significantly affects MICE performance. Combining MICE with stronger predictive algorithms, such as XGBoost, can further improve imputation accuracy and reliability (Alzubi, 2023; Alzubi et al., 2020; Khawaja et al., 2023).

This paper proposes a novel method that integrates the XGBoost model with MICE (XGBoost-MICE) to address missing data in mine ventilation parameters. The XGBoost model is trained on ventilation parameters to predict missing values, while MICE generates multiple imputed datasets to enhance the reliability and accuracy of the imputation results. Experimental results show that the XGBoost-MICE method significantly improves imputation accuracy compared to traditional methods. The innovation of this study lies in combining XGBoost with MICE to provide a robust solution for handling missing data in mine ventilation parameters, offering a new perspective and technical support for the intelligent management of mine ventilation systems.

## 2 XGBoost-based multiple imputation by chained equations regression method

### 2.1 XGBoost

XGBoost (Extreme Gradient Boosting) is an efficient ensemble learning method widely used in regression, classification, and ranking tasks. Based on the Gradient Boosting Trees (GBT) algorithm, XGBoost builds multiple weak learners (decision trees) and combines them into a strong learner to model complex data accurately. In missing data imputation tasks, XGBoost leverages the ensemble of multiple trees to predict missing values precisely, overcoming the limitations of traditional methods when handling complex data. The model is trained by minimizing a loss function and improved iteratively through weighted adjustments (Zhang et al., 2023; Qiu et al., 2022).

The core objective of XGBoost is to incrementally optimize the loss function by constructing decision trees iteratively. The loss function includes a regularization term to control model complexity and avoid overfitting. Through this approach, XGBoost effectively captures nonlinear relationships among features, making it highly suitable for predicting missing values in datasets with intricate dependencies. As shown in Equation (1):

(1) $$L\left(\theta \right)=\overset{n}{\underset{i=1}{\sum }}𝓁\left(y_{i},{\hat{y}}_{i}\right)+\overset{K}{\underset{k=1}{\sum }}\Omega \left(f_{k}\right)$$

In this context, $𝓁\left(y_{i},{\hat{y}}_{i}\right)$ represents the loss function, which quantifies the error between the predicted value ${\hat{y}}_{i}$ and the true value $y_{i}$ . Commonly used loss functions include Mean Squared Error (MSE) and logarithmic loss, among others.

The term $\Omega \left(f_{k}\right)$ denotes the regularization term for controlling the complexity of the decision tree. This term helps prevent overfitting by penalizing overly complex models. A typical regularization form is given as shown in Equation (2):

(2) $$\Omega \left(f_{k}\right)=\gamma T+\frac{1}{2}\lambda {\theta_{k}}^{2}$$

Here, T is the number of leaf nodes in the tree, *γ* is a parameter that penalizes the number of leaf nodes, *λ* is a regularization parameter, and $\theta_{k}$ represents the weights of the leaf nodes. This regularization encourages simpler models while maintaining prediction accuracy.

When applied to the processing of missing mine ventilation parameters, XGBoost can learn the relationships between different ventilation parameters, such as wind speed and pressure, from the training data and predict the missing parameter values based on these relationships. In this way, XGBoost not only efficiently handles missing data but also preserves the complex nonlinear relationships between variables during the process, ensuring high accuracy in the imputation results. In the task of imputing mine ventilation parameters, the regression model of XGBoost can be expressed as shown in Equation (3) (Zhu et al., 2021):

(3) $${\hat{y}}_{i}=\overset{K}{\underset{k=1}{\sum }}f_{k}\left(x_{i}\right)$$

Where ${\hat{y}}_{i}$ is the predicted value for the *i*-th sample, $x_{i}$ is the input feature of the *i*-th sample, $f_{k}\left(x_{i}\right)$ is the output of the k-th tree model, and K is the number of trees. By training the XGBoost model, the predicted value ${\hat{y}}_{i}$ for each missing sample can be obtained, and these predicted values can be used to fill in the missing data of the mine ventilation parameters.

### 2.2 Mice

Chain Multiple Imputation by Chained Equations (MICE) is a widely used statistical method for handling missing data. The MICE method constructs multiple regression models and uses an iterative approach to impute the missing values of each variable, generating multiple imputation results that enhance the accuracy and reliability of the imputation (Ni et al., 2024). This method is widely applied to data imputation in areas such as mine ventilation parameters, as mine ventilation data often exhibit multiple missing values and complex correlations, which MICE can effectively handle.

**Figure 1:**
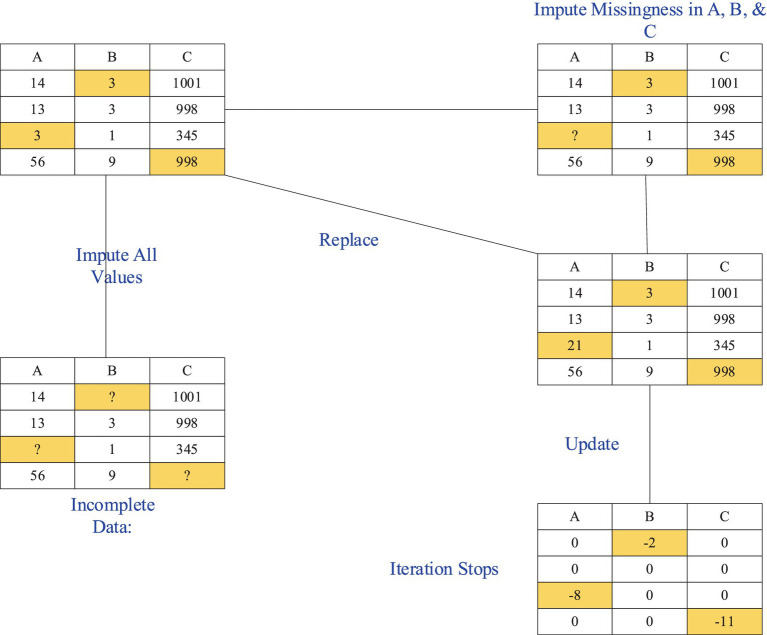
The incomplete data and imputation process based on the chain multiple imputation regression method.

The basic concept of MICE is to iteratively impute the missing values of each variable. For each variable with missing values, MICE uses other variables as predictors to build a regression model to predict the missing values, and then inserts the predicted values into the data (Samad et al., 2022). The predictions for other missing values are updated based on the imputed data until convergence, as shown in Figure 1. Specifically, the process of MICE can be summarized in the following steps:

Initial imputation: For each missing value variable Yj, an initial imputation value Yj(0) can be obtained using simple imputation methods (such as mean imputation or regression imputation).

Iterative imputation: In the t-th iteration, the missing value variable Yj is predicted using the imputed values of other variables. As shown in Equation (4):

(4) $$Y_{j}^{\left(t\right)}=f_{j}\left(X_{1}^{\left(t\right)},X_{2}^{\left(t\right)},\ldots ,X_{p}^{\left(t\right)}\right)$$

Where X_1_,X_2_,…,X_p_ are the known variables related to Y_j_, and f_j_ is the regression model for Y_j_.

Repeated iteration: The missing values are updated through multiple iterations until the imputation results for all variables converge, meaning no significant changes occur (Alruhaymi and Kim, 2021). The estimates after combining multiple imputation results can be calculated using the following Equation (5):

(5) $$\hat{\theta }=\frac{1}{m}\overset{m}{\underset{i=1}{\sum }}{\hat{\theta }}^{\left(i\right)}$$

Where ${\hat{\theta }}^{\left(i\right)}$ is the model parameter estimated based on the *i*-th imputation result.

For the imputation of missing mine ventilation parameters, the MICE method treats the missing ventilation parameters as target variables, using other known ventilation data (such as wind speed, pressure, temperature, etc.) as predictor variables to construct a regression model for imputation. Each regression model can be expressed as shown in Equation (6):

(6) $$Y_{j}=f\left(X_{1},X_{2},\ldots ,X_{p}\right)+\in$$

Where $Y_{j}$ is the missing value of the j-th mine ventilation parameter, $X_{1},X_{2},\ldots ,X_{p}$ are the known ventilation parameters related to it, and *ϵ* is the error term.

### 2.3 Chain multiple imputation regression based on the XGBoost model

**Figure 2:**
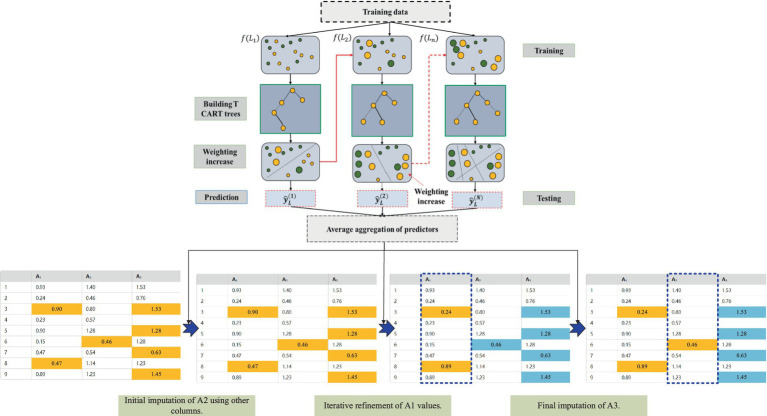
Chain equation multiple imputation method based on XGBoost (Giuliani et al., 2021).

In the process of handling missing mine ventilation parameter data, the Chain Multiple Imputation Regression method based on the XGBoost model (XGBoost-MICE) efficiently fills in missing values, ensuring the completeness and accuracy of ventilation system data (Giuliani et al., 2021). This method generates multiple imputation results through an iterative process and uses the XGBoost model to predict each missing value. The process is shown in Figure 2. The specific steps are as follows:

#### 2.3.1 Step 1: Initialize random imputation and obtain complete dataset

First, for the mine ventilation parameter dataset with missing values, we generate the initial complete dataset, Rnd_Dataset, through random imputation. For each missing value, some data points from the known values of the current attribute are randomly selected to fill in the missing value. The generated Rnd_Dataset will be used in the subsequent imputation process.

#### 2.3.2 Step 2: XGBoost-based prediction and imputation (iterative process)

**Figure 3:**
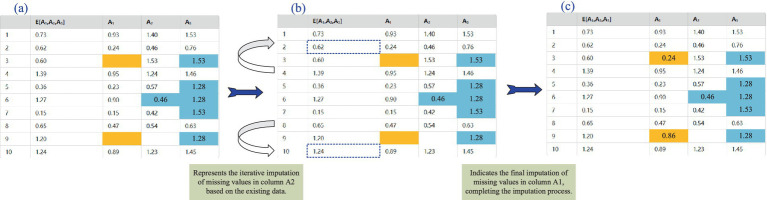
Prediction mean matching process based on XGBoost. **(a)** Transform data features; **(b)** Predict similar data; **(c)** Data imputation.

Next, for the initial complete dataset Rnd_Dataset, we begin the multiple iterations (with the number of iterations set to n). In each iteration, for each missing value attribute (such as ventilation wind speed, temperature, pressure, etc.), the XGBoost model is used to make predictions. The specific operation is as follows:

As shown in Figure 3, for each missing value attribute (for example, A1,A2,…), other known attributes are used as the feature matrix. For instance, the prediction of the missing value of A1 may depend on the known values of A2 and A3.

- The XGBoost model is trained on these feature matrices to predict the missing attribute’s value. The basic form of the XGBoost model is following Equation (7):
(7) $${\hat{y}}_{i}=\overset{K}{\underset{k=1}{\sum }}f_{k}\left(x_{i}\right)$$

Once the predicted result E[A1∣A2,A3] is obtained, imputation is performed based on the matching relationship between the predicted values and the known data in the original dataset. During the imputation process, original data values that are close to the predicted values for the missing value are selected for imputation. For example, if the predicted values for A1 are 0.60 and 1.20, the known data will be searched to find samples that are close to these values, and the missing A1 attribute will be imputed based on these sample values.

To further refine the imputation, the parameter mean_match_candidate is set to limit the number of candidate data points used for imputation. For instance, if mean_match_candidates = 5, the 5 closest values to the missing value prediction are selected, and their mean is calculated to serve as the final imputed result.

For each missing value attribute, the above steps are repeated until all missing values for the attribute are filled, resulting in a complete dataset.

#### 2.3.3 Step 3: Repeat the iterative process until stopping conditions are met

Once the imputation after the first iteration is completed, the imputed complete dataset is used again to perform Step 2. Through multiple iterations, missing values are further imputed and optimized until the maximum number of iterations n is reached. Each iteration generates a more accurate dataset, progressively eliminating the impact of missing data.

#### 2.3.4 Step 4: Generate multiple complete datasets and perform final analysis

By executing Steps 1 through 3, we can generate m different complete datasets. Each dataset is derived from different initial imputation values and iteration processes, providing a certain level of diversity. Finally, these m complete datasets are analyzed to produce the final imputation results. To combine the multiple imputation results, the following formula is used for aggregation, as shown in Equation (8):

(8) $$\hat{\theta }=\frac{1}{m}\overset{m}{\underset{i=1}{\sum }}{\hat{\theta }}^{\left(i\right)}$$

The step-by-step implementation of the XGBoost-MICE method is detailed in Table 1, outlining each function’s role in the imputation workflow.

**Table 1:** XGBoost-MICE imputation process.

Step	Procedure	Function used
Step 1	Initialize dataset with missing values and simulate missing data for controlled experiments.	missing_data_generate()
Step 2	Perform initial random imputation to fill missing values as placeholders.	–
Step 3	Iterate through each variable with missing values, treating it as the dependent variable and using other variables as predictors.	mice()
Step 4	Train an XGBoost model on the available data to predict missing values for the target variable.	XGBoost()
Step 5	Replace missing values with predictions from XGBoost while maintaining dataset structure.	–
Step 6	Repeat the imputation process for all missing variables iteratively until convergence is achieved.	mice()
Step 7	Generate multiple complete datasets with different imputation iterations.	with()
Step 8	Pool the multiple imputed datasets and aggregate the final imputation results.	pool()
Step 9	Validate the imputed dataset using statistical metrics such as MSE, MAE, and R^2^.	–
Step 10	Output the final complete dataset for further analysis or modeling.	–

## 3 Mine ventilation parameter missing data imputation experiment

The dataset consists of historical ventilation data from the Shangwan Coal Mine, including various ventilation parameters such as airflow, wind speed, temperature, and pressure, measured at different points in time across multiple mine tunnels. The dataset comprises 312 samples, each representing a set of ventilation measurements collected from one of the mine tunnels at a specific time.

### 3.1 Mine ventilation parameter missing data imputation process

The process of imputing missing mine ventilation parameter data using the XGBoost-based Chain Multiple Imputation Regression method (XGBoost-MICE) consists of three stages (Pan et al., 2022):

#### 3.1.1 Step 1: XGBoost-MICE function to impute missing data

In this stage, the with() function is used to process the imputed mine ventilation parameter data. The with() function accepts the imputed datasets and applies statistical methods or machine learning models to analyze or refine them. The function generates output, which is used for further evaluation or integration into the next step of the imputation process.

#### 3.1.2 Step 2: Data analysis

Once the data has been imputed, the pool() function is used. The pool() function takes in multiple imputed datasets, each generated from different initial imputations and iterations. These multiple datasets are then combined to form a single dataset, ensuring that the final imputed dataset reflects the diversity and reliability of the imputation process.

#### 3.1.3 Step 3: Results integration

In the final stage, the pool function integrates the multiple analysis results from Step 2 according to an optimal principle, obtaining the final imputed results for the mine ventilation parameters with missing data. By combining the analysis results of multiple imputed datasets, the accuracy and reliability of the imputed results can be further improved. The final integrated results will more accurately reflect the parameter relationships within the mine ventilation system, providing precise data support for intelligent management systems.

**Figure 4:**
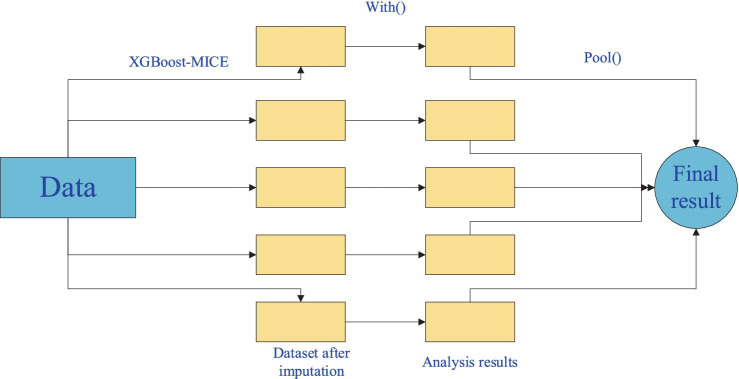
Mine ventilation parameter missing data imputation process.

The mine ventilation parameter missing data imputation process is shown in Figure 4. This process, through multiple imputations and data analyses, ensures that the final dataset is more complete and can provide accurate data support for the optimization and adjustment of the mine ventilation system.

### 3.2 Evaluation metrics

For linear regression and other regression models, evaluation of continuous, well-fitting data cannot be done using evaluation metrics for discrete binary classifiers. Therefore, metrics such as Mean Squared Error (MSE), Mean Absolute Error (MAE), Explained Variance, and Coefficient of Determination (R^2^) are introduced to measure the performance of the model (Asselman et al., 2023; Li, 2022; Osman et al., 2021). These metrics can be directly computed using functions from the sklearn library in Python.

1. Mean Squared Error (MSE): This metric calculates the mean of the squared differences between the fitted data and the original data at the corresponding sample points. It is used to assess how well the fitted data matches the original true values. A smaller MSE value indicates a better fit. The formula for calculating MSE is following Equation (9):
(9) $$MSE=\frac{1}{N}\overset{N}{\underset{i=1}{\sum }}{\left(y_{i}-{\hat{y}}_{i}\right)}^{2}$$

In the formula, N is the number of samples; iii represents each data sample; $y_{i}$ is the original true value; and ${\hat{y}}_{i}$ is the predicted value.

2. Explained Variance (Explained Variance, Evar): This metric measures how closely the spread of the differences between all predicted values and the samples matches the spread of the samples themselves. The value of Evar ranges from 0 to 1, with a higher value indicating that the spread of the predicted values is closer to the spread of the samples, and a lower value indicating poorer performance. The formula for calculating explained variance is following Equation (10):
(10) $$E_{var}\left(y,\hat{y}\right)=1-\frac{\frac{1}{N}\overset{N}{\underset{i=1}{\sum }}{\left(z_{i}-{\bar{z}}_{i}\right)}^{2}}{\frac{1}{N}\overset{N}{\underset{i=1}{\sum }}{\left(y_{i}-{\bar{y}}_{i}\right)}^{2}}$$

In the formula, ${\bar{y}}_{i}$ is the mean of the original values; $z_{i}$ represents the difference between the sample values and the predicted values; and ${\bar{z}}_{i}$ is the mean of the sample values.

3. Coefficient of Determination (*R*^2^): The *R*^2^ metric measures how well the model fits the data by representing the proportion of variance in the dependent variable that is predictable from the independent variables. The formula for calculating *R*^2^ is following Equation (11):
(11) $$R^{2}=1-\frac{\overset{N}{\underset{i=1}{\sum }}{\left(y_{i}-{\hat{y}}_{j}\right)}^{2}}{\overset{N}{\underset{i=1}{\sum }}{\left(y_{i}-{\bar{y}}_{i}\right)}^{2}}$$
Mean Absolute Error (MAE): This metric calculates the average of the absolute errors between the predicted values and the true values. It is used to assess how close the predicted results are to the true data values. A smaller MAE value indicates a better fit. The formula for calculating MAE is following Equation (12):
(12) $$E_{MA}=\frac{1}{N}\overset{N}{\underset{i=1}{\sum }}|y_{i}-{\hat{y}}_{i}|$$

### 3.3 Shawan coal mine ventilation imputation experiment

#### 3.3.1 Mine ventilation parameter dataset

Due to the complex and variable underground environment, obtaining complete and accurate sample data from only a few measurements is challenging. Therefore, the historical measurement data from the Shawan Coal Mine’s ventilation system is used to address the missing mine ventilation parameters. Based on the preliminary formation of the mine ventilation system, a fixed portion of the airflow is adjusted and allocated through repeated tuning until a complete dataset of mine ventilation parameters, consistent with the on-site conditions, is obtained. This dataset includes various ventilation parameters, such as wind speed, air volume, temperature, pressure, etc., and is recorded in an Excel spreadsheet, as shown in Table 2. By handling the missing values in this data and applying the Chain Multiple Imputation by Chained Equations method based on the XGBoost model (XGBoost-MICE), a complete dataset of mine ventilation parameters was obtained.

**Table 2:** Mine ventilation parameter dataset.

Tunnel ID	Cross-sectional area (m^2^)	Section air flow (m^3^/s)	Section wind speed (m/s)	Actual resistance difference (Pa)	Friction wind resistance per 100 meters (N·s^2^·m^−8^)
1	13.74	33.53	9.66	94	0.015508
2	11.35	67.46	7.34	238	0.008113
3	13.61	71.88	6.24	97	0.013640
4	14.77	18.68	5.66	243	0.009396
5	10.68	63.22	10.05	145	0.009295
6	7.93	33.87	4.01	169	0.008389
7	8.90	55.47	9.89	151	0.008728
8	11.16	32.21	4.86	208	0.010526
9	13.17	21.80	3.91	23	0.011498
10	8.86	48.32	6.80	56	0.009646
21	9.97	21.39	2.58	76	0.012893
22	8.23	35.52	7.83	243	0.009236
23	13.23	52.73	4.21	58	0.013176
24	14.55	42.15	8.67	271	0.007781
25	10.34	57.28	9.04	102	0.010145
26	12.47	28.69	3.65	134	0.011735
27	14.06	44.13	5.77	153	0.014112
28	10.78	19.55	6.92	125	0.012834
29	9.15	23.68	4.76	220	0.008954
30	11.86	62.24	10.12	87	0.015108
31	14.09	15.74	6.30	94	0.013294
32	7.91	33.65	9.81	212	0.010827
33	9.35	46.29	5.49	159	0.008819
34	11.41	64.56	7.94	143	0.011946
35	12.90	38.74	6.11	69	0.012564
36	10.28	48.94	4.82	241	0.009306
37	13.45	54.88	8.34	173	0.014715
38	14.71	30.42	7.43	136	0.009954
39	12.30	26.37	3.89	108	0.012384
40	9.60	50.87	6.99	193	0.010847
**…**					
304	13.54	36.12	9.24	91	0.014093
305	11.28	27.34	4.98	114	0.009817
306	9.44	41.09	7.56	168	0.008904
307	7.88	14.54	5.24	203	0.010193
308	14.02	32.68	9.64	185	0.012783
309	10.72	24.72	6.18	131	0.011524
310	8.52	55.10	8.43	126	0.009422
311	11.97	38.26	10.04	157	0.012214
312	13.82	19.58	3.97	217	0.014716
313	9.50	29.73	5.89	178	0.008712

By conducting on-site measurements of the Shawan Coal Mine’s ventilation system and collecting relevant data, a ventilation simulation model for the mine was constructed using these measurements. Through airflow distribution and adjustment, a complete dataset of mine ventilation parameters was ultimately obtained. Next, the XGBoost-MICE method was applied to impute the missing values in this dataset, generating multiple imputed datasets to ensure the accuracy and reliability of the imputation results. Finally, the imputed datasets were compared with the original dataset to verify the feasibility and accuracy of the imputation method.

#### 3.3.2 Imputation experiment and result analysis

To validate the feasibility and accuracy of the model in imputing missing mine ventilation parameter data, a custom function was used to randomly introduce missing values into the original complete dataset, simulating the random missing characteristics of actual mine ventilation parameter data. By adjusting the parameters in the custom function, different missing attributes, missing proportions, and iteration counts were set, and three sets of experiments were designed. In the experiments, attributes such as “cross-sectional area” and “section air volume” were designated as the missing columns. The results showed that when the number of iterations reached 30, the model had essentially converged. To avoid the impact of iteration counts on the experimental results, the iteration count was fixed at 30 for both Experiment 1 and Experiment 2, while the effect of iteration count on the results was separately explored in Experiment 3.

**Figure 5:**
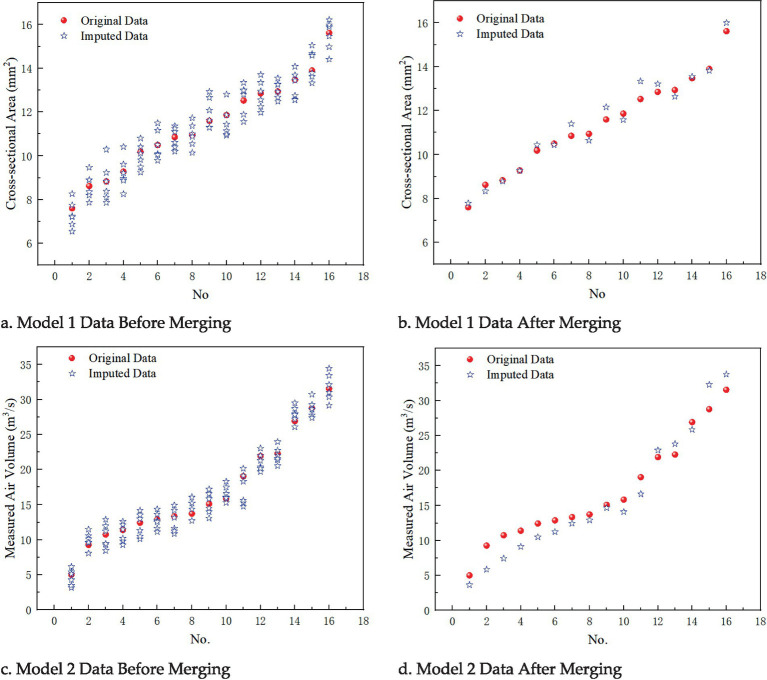
Imputation effects before and after data merging for models 1 and 2. **(a)** Model 1 data before merging; **(b)** Model 1 data after merging; **(c)** Model 2 data before merging; **(d)** Model 2 data after merging.

In Experiment 1, the custom function missing_data_generate() was used to set the missing_columns parameter, selecting “cross-sectional area” and “section air volume” as the missing columns for imputation testing. Specifically, the following models were used: • Model 1: The missing column is “cross-sectional area,” with a missing data ratio of 5% and 30 iterations. • Model 2: The missing column is “section air volume,” with a missing data ratio of 5% and 30 iterations. The comparison of the imputed datasets for Models 1 and 2, before and after merging the analysis, is shown in Figure 5.

**Figure 6:**
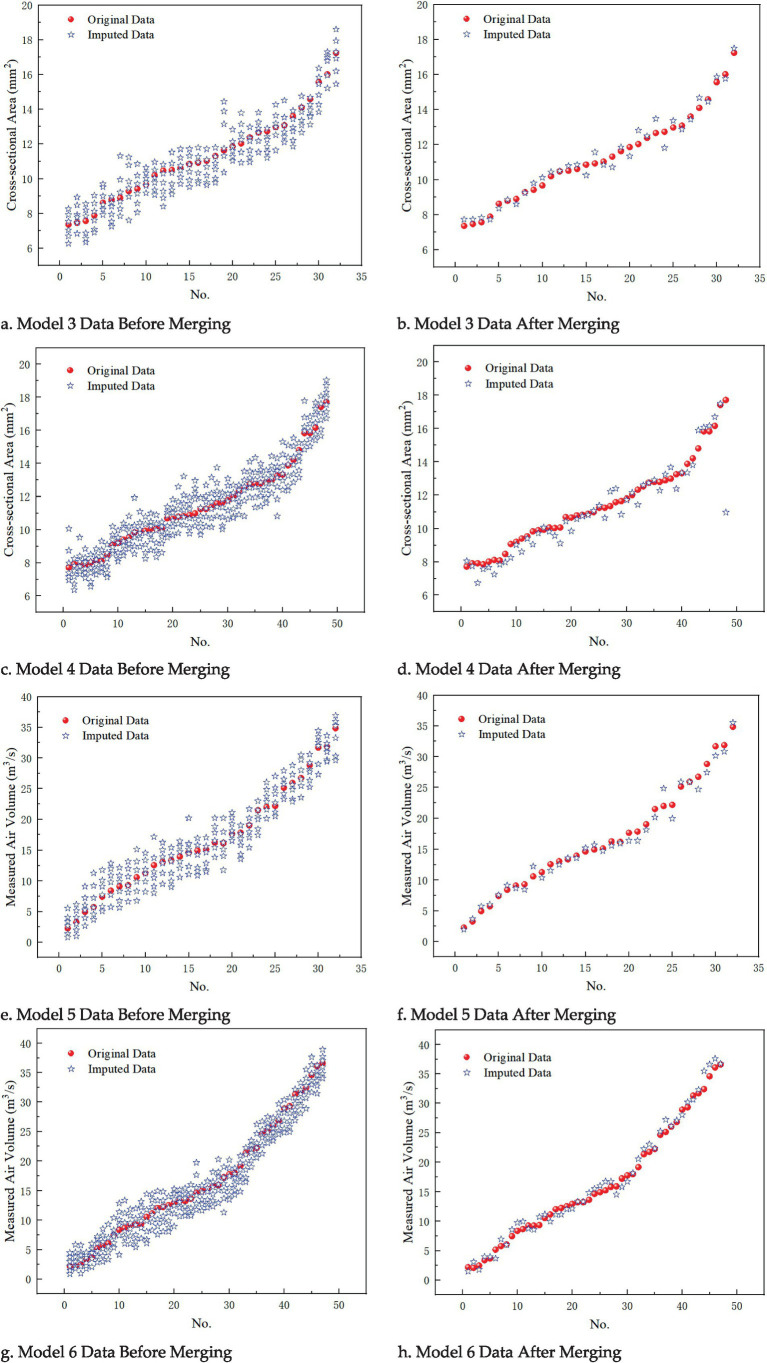
Imputation effects before and after merging datasets for models 3, 4, 5 and 6. **(a)** Model 3 data before merging; **(b)** Model 3 data after merging; **(c)** Model 4 data before merging; **(d)** Model 4 data after merging; **(e)** Model 5 data before merging; **(f)** Model 5 data after merging; **(g)** Model 6 data before merging; **(h)** Model 6 data after merging.

In Experiment 2, the custom function missing_data_generate() was used to set the missing data proportion through the missing_rate parameter. Taking “cross-sectional area” and “section air volume” as examples, missing rates of 10 and 15% were set. The results can be compared with those from Experiment 1. Specifically: • Model 3: The missing column is “cross-sectional area,” with a missing rate of 10%, and the number of iterations is set to 30. • Model 4: The missing column is still “cross-sectional area,” but the missing rate is increased to 15%, with the number of iterations remaining at 30. • Model 5: The missing column is “section air volume,” with a missing rate of 10%, and the number of iterations is set to 30. • Model 6: The missing column is “section air volume,” with a missing rate of 15%, and the number of iterations is set to 30. The comparison of the imputed data for Models 3 to 6, before and after merging the analysis, is shown in Figure 6.

In Experiment 3, the number of iterations during the imputation process was set using the iteration parameter of the imputation function mice(). In this case, “friction wind resistance per 100 meters” was chosen as an example, and the final iteration count was set to 50. A program was written to record the changes in the mean value of the data after each iteration. The specific model settings are as follows: • Model 7: The missing column is “friction wind resistance per 100 meters,” with a missing rate of 10% and 30 iterations. • Model 8: The missing column is still “friction wind resistance per 100 meters,” with a missing rate of 10%, but the iteration count is increased to 50.

**Figure 7:**
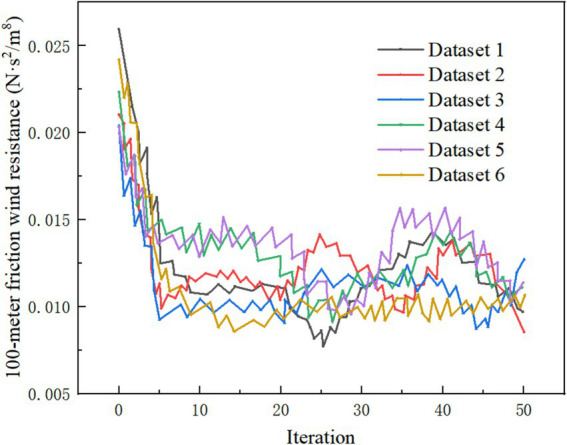
Mean convergence illustration.

By outputting the mean of the data after each iteration, the convergence of the mean values is shown in Figure 7. To further verify the stability of the imputation results, datasets = 6 was set, meaning six complete datasets were generated simultaneously during the imputation process. The comparison of the mean value changes across each complete dataset shows that when the iteration count reached 6, the data mean had already converged. After further increasing the number of iterations, the data mean tended to stabilize. This leads to the conclusion that an appropriate number of iterations ensures the accuracy of the imputation results, while excessive iterations have little impact on the convergence of the mean.

**Figure 8:**
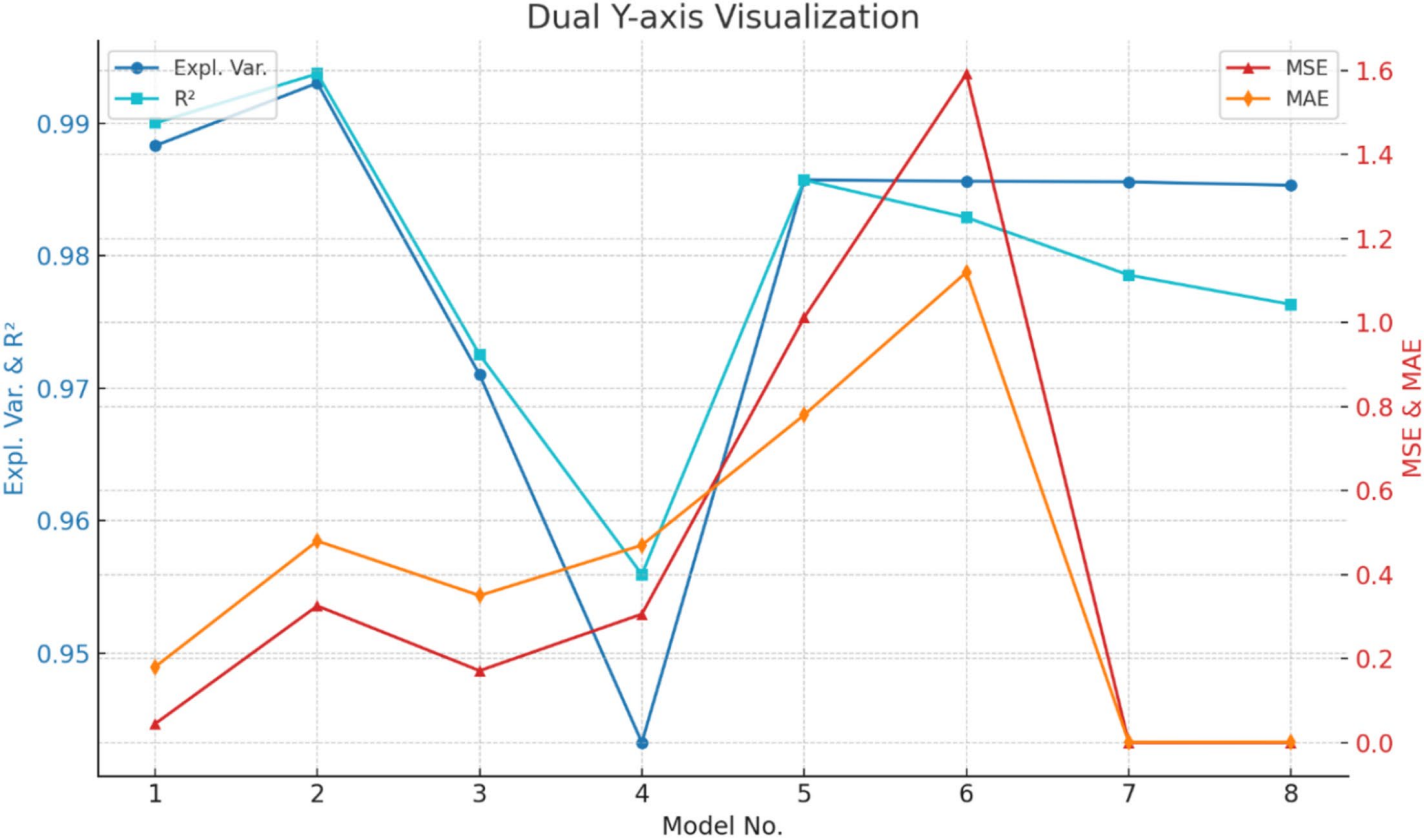
Model evaluation standard illustration.

According to the three sets of experiments, the evaluation standards for the mine ventilation parameter missing data completion model for Shawan Coal Mine are shown in Figure 8.

Through the analysis of the imputation results and evaluation metrics for Models 1 to 6, it was found that when the missing rate of the “cross-sectional area” data increased from 5 to 15%, the Mean Squared Error (MSE) rose from 0.0445 to 0.3254, an increase of 0.2614, indicating a significant impact of the higher missing rate on imputation accuracy. Meanwhile, the Explained Variance decreased from 0.988309 to 0.943267 (a decrease of 0.045), and the Coefficient of Determination (R^2^) dropped from 0.990019 to 0.955951 (a decrease of 0.035), suggesting that the model’s explanatory power and fit declined as the missing rate increased. Additionally, the Mean Absolute Error (MAE) increased from 0.180 to 0.470, an increase of 0.29, further indicating that a higher missing rate significantly increases the imputation bias.

Similarly, when the missing rate of the “section air volume” data increased from 5 to 15%, the MSE increased significantly from 1.0121 to 1.5924, an increase of 1.267. The Explained Variance only decreased from 0.985740 to 0.985635 (a decrease of 0.00741), and R^2^ decreased from 0.985721 to 0.982897 (a decrease of 0.0108). This indicates that while the high missing rate has a major impact on the MSE and MAE, its effect on the Explained Variance and R^2^ is minimal. At the same time, the MAE increased from 0.780 to 1.120, an increase of 0.64, further showing that a higher missing rate significantly affects imputation errors.

The iteration count test for the “friction wind resistance per 100 meters” attribute showed that when the number of iterations was 30, the MSE, Explained Variance, and R^2^ had already stabilized, indicating that the imputation results had reached a good convergence state. Increasing the number of iterations to 50 did not lead to significant changes, further confirming the model’s convergence and stability within a reasonable number of iterations. This suggests that, in practical applications, setting a reasonable number of iterations can ensure the accuracy of the imputation results while improving computational efficiency.

A comprehensive analysis shows that the imputation method proposed in this study demonstrates high accuracy and reliability across different missing attributes and missing rates. Under low missing rates, the model’s imputation errors are small. As the missing rate increases, the imputation error and bias of the predicted data increase, but the overall performance remains relatively stable. Meanwhile, with a reasonable number of iterations, the imputation method exhibits good convergence and adaptability, providing reliable technical support for the imputation and analysis of mine ventilation data.

## 4 Discussion

### 4.1 Limitations and future research directions

The results of this study highlight the need to comprehensively consider missing rates, data characteristics, and algorithm performance when imputing missing data in mine ventilation parameters. Our experiments revealed that the model performed optimally under low missing rates (5%), with minimal MSE and MAE values. However, as the missing rate increased, imputation accuracy notably declined. For instance, in the “cross-sectional area” scenario, MSE increased by 0.2614 and MAE by 0.29, showing a significant impact of higher missing rates on imputation results. Experiments with different attributes demonstrated that the model retained stability even under high missing rates, particularly for the “airflow volume” attribute, where the R-squared dropped by only 0.0108, indicating the model’s adaptability to complex attributes. Additionally, iteration experiments validated that a moderate number of iterations (e.g., six) ensured accurate imputation while improving computational efficiency. But we recognize that it is important to acknowledge its limitations for a more balanced perspective. One limitation is its performance under extremely high missing rates (greater than 15%), where imputation accuracy may decline significantly. While our experiments have demonstrated the method’s effectiveness up to 15% missing data, further testing is needed to assess its robustness in scenarios with higher missing rates. Additionally, the method’s applicability to non-linear or non-stationary data is another area that warrants further investigation. The current study primarily focused on data with relatively stable relationships among parameters, and future work should explore how the model adapts to data exhibiting non-stationary behavior or extreme volatility. Another limitation is the computational intensity of XGBoost, which may pose challenges in large-scale or real-time applications. The study did not address these concerns in detail, but future research should investigate strategies to improve computational efficiency, such as parallel processing or optimized implementations, without compromising the imputation accuracy. These aspects will help determine the practical feasibility of deploying XGBoost-MICE in real-world, large-scale applications.

This study demonstrates the effectiveness of the XGBoost-MICE method, but we acknowledge that a clearer comparison with existing imputation techniques would provide more context for its advantages. Compared to traditional imputation methods like mean imputation and k-Nearest Neighbors (k-NN), which are simple and widely used, XGBoost-MICE offers several key improvements. While mean imputation fills missing values based on the average of known data, and k-NN estimates missing values based on the closest available data points, these methods can struggle when dealing with complex datasets with non-linear relationships. In contrast, XGBoost-MICE integrates the powerful predictive capabilities of XGBoost, which captures complex, non-linear dependencies in the data, with the iterative and multiple imputations process of MICE, which reduces bias and enhances the reliability of imputed results. This hybrid approach enables more accurate and robust imputation, especially in datasets with intricate patterns of missingness.

However, it is important to note that XGBoost is computationally intensive, which raises concerns regarding its runtime, memory usage, and feasibility for large-scale or real-time applications. The study did not address these aspects in detail. Future studies should examine these factors more thoroughly, considering how the model’s performance can be optimized for real-time or large-scale applications. Furthermore, research into parallel processing or more efficient implementation techniques could be explored to reduce computational costs without sacrificing imputation accuracy.

While various missing data imputation methods, such as XGBoost and MICE, have been widely used to handle missing values, each has limitations. XGBoost, as a powerful ensemble learning model, can effectively predict missing values by capturing complex relationships in the data. However, it may not fully account for the variability in imputation results caused by the randomness of missing data. In contrast, MICE, a classic multiple imputation method, generates multiple imputed datasets through iterative regression models, which reduces bias but lacks the predictive power needed for handling complex datasets (Shah et al., 2014; Mera-Gaona et al., 2021; Zhang, 2016). The integration of XGBoost with MICE in the proposed method enhances both the accuracy and stability of imputation results, as it combines the strong predictive capabilities of XGBoost with the reliability of multiple imputations from MICE. This hybrid approach not only improves imputation accuracy for mine ventilation parameters but also provides a robust solution for data completeness in real-world scenarios, especially in settings with varying missing rates and complex correlations.

In real-world scenarios, the method can be integrated into mine ventilation control systems to optimize airflow distribution and ensure safety. For example, by imputing missing ventilation parameters, the method can provide accurate data for predictive modeling, enabling operators to adjust ventilation systems in real-time based on the predicted air quality and ventilation requirements. This approach can significantly reduce the reliance on manual adjustments and enhance the overall efficiency of the ventilation system. In addition, the XGBoost-MICE method could be deployed in conjunction with IoT (Internet of Things) sensors in mining operations. These sensors continuously monitor ventilation parameters such as airflow, temperature, and pressure, providing real-time data to the system. When data is missing due to sensor malfunctions or communication failures, the XGBoost-MICE method can impute the missing values, ensuring uninterrupted operation of the ventilation system. The integration of this method into smart mining systems would improve decision-making, reduce downtime, and contribute to safer working environments by maintaining optimal ventilation conditions.

### 4.2 Comparison with k-nearest neighbors (k-NN) model

The effectiveness of the XGBoost-MICE method in imputing missing mine ventilation parameters can be assessed by comparing it with the k-Nearest Neighbors (k-NN) model, a widely used imputation technique. The k-NN algorithm estimates missing values by identifying k similar data points based on a distance metric, commonly Euclidean distance, and computing the mean or weighted mean of their corresponding values. While k-NN performs well in datasets where similar patterns exist locally, it exhibits several limitations in handling complex and high-dimensional data.

The computational complexity of k-NN increases with the dataset size because it requires distance calculations for every missing data point against all available data. As the number of dimensions increases, the curse of dimensionality leads to degraded performance, reducing the reliability of imputations. k-NN is highly sensitive to the chosen number of neighbors, as smaller k values may introduce high variance while larger k values may smooth out meaningful variations. Moreover, k-NN assumes that missing values can be inferred based on the proximity of known samples, which may not hold in datasets where missingness is not random or exhibits strong correlations between attributes.

In contrast, XGBoost-MICE applies a tree-based ensemble learning method to model complex relationships within the data. XGBoost captures non-linear dependencies and interactions between variables, offering greater accuracy in high-dimensional datasets. Unlike k-NN, which relies solely on local similarity, XGBoost learns predictive patterns from the entire dataset, leading to more stable and consistent imputations. The iterative nature of MICE further refines the imputation results, reducing bias and ensuring that variability in missing data is adequately accounted for.

**Figure 9:**
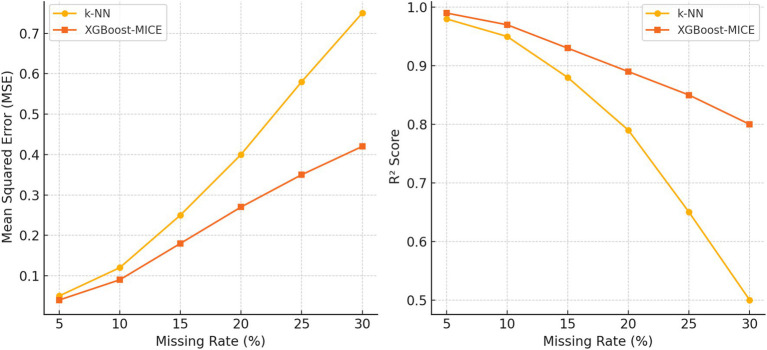
Comparison of imputation accuracy between k-NN and XGBoost-MICE under different missing rates.

Figure 9 presents a comparative analysis of imputation accuracy between k-NN and XGBoost-MICE under different missing rates. When the missing rate is below 10%, k-NN produces reasonable imputations with a mean squared error (MSE) comparable to XGBoost-MICE. As the missing rate increases beyond 10%, k-NN’s performance declines significantly, while XGBoost-MICE maintains higher accuracy. The reduction in explained variance and coefficient of determination (R^2^) is more pronounced in k-NN, indicating that its imputation quality deteriorates under higher missing rates.

**Figure 10:**
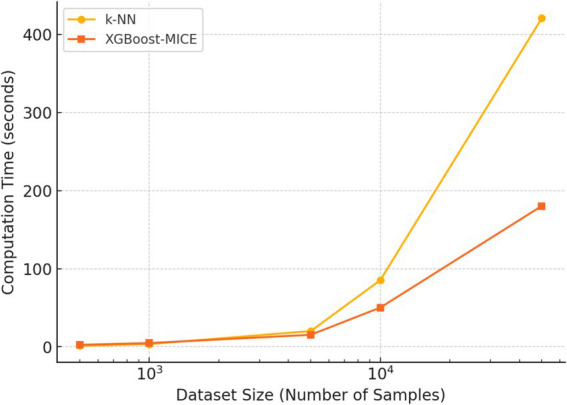
Computation time comparison between k-NN and XGBoost-MICE for different dataset sizes.

The time complexity of both methods is also evaluated. Figure 10 illustrates the computation time required for different dataset sizes. k-NN requires exponentially longer processing time as the dataset grows, while XGBoost-MICE maintains a more efficient scaling pattern.

## 5 Conclusion

This study proposed an XGBoost-MICE (Multiple Imputation by Chained Equations) regression method to address missing data in mine ventilation parameters, demonstrating its effectiveness and stability through experimental validation. Results revealed that the method achieved high imputation accuracy under low missing rates (5%), with minimal Mean Square Error (MSE) and Mean Absolute Error (MAE). As missing rates increased, the accuracy of imputation decreased, but the model retained overall stability. For complex attributes such as “cross-sectional area,” the impact of higher missing rates on R-squared and Explained Variance was minimal, indicating the algorithm’s adaptability to complex data. Iteration experiments confirmed the convergence and efficiency of the method, showing that reasonable iteration numbers significantly improve imputation reliability. In conclusion, the proposed method offers a novel technical approach for addressing missing data issues in mine ventilation systems and provides robust support for intelligent optimization and safe production in mines.

## Data Availability

The original contributions presented in the study are included in the article/supplementary material, further inquiries can be directed to the corresponding author.
